# Water Management Impacts on Chromium Behavior and Uptake by Rice in Paddy Soil with High Geological Background Values

**DOI:** 10.3390/toxics11050433

**Published:** 2023-05-05

**Authors:** Zeting Guan, Ran Wei, Ting Liu, Jingjing Li, Ming Ao, Shengsheng Sun, Tenghaobo Deng, Shizhong Wang, Yetao Tang, Qingqi Lin, Zhuobiao Ni, Rongliang Qiu

**Affiliations:** 1 Guangdong Laboratory for Lingnan Modern Agriculture, Guangdong Provincial Key Laboratory of Agricultural & Rural Pollution Abatement and Environmental Safety, College of Natural Resources and Environment, South China Agricultural University, Guangzhou 510642, China; 2 Guangdong Provincial Key Laboratory of Environmental Pollution Control and Remediation, School of Environmental Science and Engineering, Sun Yat-sen University, Guangzhou 510006, China; 3 Institute of Quality Standard and Monitoring Technology for Agro-Products of Guangdong Academy of Agricultural Sciences, Guangzhou 510640, China

**Keywords:** chromium, paddy soil, rice, water management, high geological background

## Abstract

Chromium (Cr) is an expression toxic metal and is seriously released into the soil environment due to its extensive use and mining. Basalt is an important Cr reservoir in the terrestrial environment. Cr in paddy soil can be enriched by chemical weathering. Therefore, basalt-derived paddy soils contain extremely high concentrations of Cr and can enter the human body through the food chain. However, the water management conditions’ effect on the transformation of Cr in basalt-derived paddy soil with high geological background values was less recognized. In this study, a pot experiment was conducted to investigate the effects of different water management treatments on the migration and transformation of Cr in a soil–rice system at different rice growth stages. Two water management treatments of continuous flooding (CF) and alternative wet and dry (AWD) and four different rice growth stages were set up. The results showed that AWD treatment significantly reduced the biomass of rice and promoted the absorption of Cr in rice plants. During the four growth periods, the root, stem and leaf of rice increased from 11.24–16.11 mg kg^−1^, 0.66–1.56 mg kg^−1^ and 0.48–2.29 mg kg^−1^ to 12.43–22.60 mg kg^−1^, 0.98–3.31 mg kg^−1^ and 0.58–2.86 mg kg^−1^, respectively. The Cr concentration in roots, stems and leaves of AWD treatment was 40%, 89% and 25% higher than CF treatment in the filling stage, respectively. The AWD treatment also facilitated the potential bioactive fractions conversion to the bioavailable fraction, compared with the CF treatment. In addition, the enrichment of iron-reducing bacteria and sulfate-reducing bacteria with AWD treatment also provided electron iron for the mobilization of Cr, thus affecting the migration and transformation of Cr in the soil. We speculated that the reason for this phenomenon may be the bioavailability of Cr was affected by the biogeochemical cycle of iron under the influence of alternating redox. This indicates that AWD treatment may bring certain environmental risks in contaminated paddy soil with high geological background, and it is necessary to be aware of this risk when using water-saving irrigation to plant rice.

## 1. Introduction

Heavy metals-contaminated agricultural soil has become a global and widespread issue [1,2], which poses a serious threat to food security. Chromium (Cr) is one of the most phytotoxic metals even at trace levels, which is released into agricultural soils through anthropogenic and geological backgrounds [3]. Cr can easily enter the food chain, and its excessive accumulation in soil–plant systems poses a risk to human health [3]. In many countries, the main natural source of Cr in agricultural soils is weathering from Cr-rich ultramafic salts (e.g., serpentine), such as France, the United States, Brazil and New Caledonia [4,5,6,7]. In China, the average content of Cr in arable land soil is 78.94 mg kg^−1^, significantly higher than the background content 57.30 mg kg^−1^. About 1.39% of arable land grain production has a high risk of Cr pollution [8]. According to the screening and control values of soil pollution risk on agricultural land, as specified in the “Soil Environmental Quality Soil Pollution Risk Control Standard (Trial)” (GB15618-2018) [9], the standard Cr concentration in paddy soil limit is 250 mg kg^−1^ (pH ≤ 5.5, 5 < pH ≤ 6.5), 300 mg kg^−1^ (6.5 < pH ≤ 7.5) and 350 mg kg^−1^ (pH > 7.5), depending on the soil pH, respectively. In basalt watersheds (such as the Yunnan and Leiqiong areas in China), the background value of Cr exceeds the national standard of China. Rice (*Oryza sativa* L.) is a staple food crop for half of the world‘s population and 60% of China‘s residents [10,11]. Therefore, it is necessary to focus on the Cr behavior in the paddy-rice system.

Rice is mostly grown under continuous flooding (CF) conditions, but due to climate change and limited development, the agronomic measure of alternative wet and dry (AWD) has been derived for the lack of water in the dry season [12]. AWD is the aerobic and anaerobic environment that manipulates rice by alternating unflooded and flooded conditions during the growing season and is an alternative to traditional continuous flooding management [13,14], which is considered to be an effective rice cultivation method. Water management in rice planting has greatly affected the redox environment of paddy soil, which can affect the bioavailability of arsenic in paddy soil and its accumulation in rice [15,16]. Similarly, as a variable valence element, Cr may lead to dynamic redox state changes under different water management conditions, thus affecting the absorption of Cr by plants [17]. It has been reported that Cr(VI) is reduced by 69–71.8% under continuous flooding conditions(CF), while only 33.3–38.6% under intermittent flooding conditions(AWD) [17]. Different chemical forms of Cr in soil can produce different biogeochemical behaviors, which is crucial for assessing the hazards of Cr pollution in soil [18,19]. Cr in soil contains exchangeable, carbonate binding, oxidizable, reducible and residual forms of Cr [20]. Oxidizable Cr and reducible Cr can also be subdivided into Fe/Mn oxides-bound fraction, organic matter-bound fraction, amorphous iron oxides-bound fraction and crystalline iron oxides bound fraction [21]. Among them, the exchangeable fraction and carbonate binding fraction of Cr are the bioavailable mobile components, and the oxidizable fraction and the reducible fraction are the potential mobile components. Although Cr in residual fraction cannot be directly absorbed by plants, it can be activated and released through hydrolysis, oxidation and reduction [22,23]. The availability and morphological changes of Cr in soil systems are also related to soil properties such as iron oxides [20,24]. Iron has a variety of oxidation and reducing states, so it can be an electron donor or acceptor in soil, and it mainly plays a role in reducing and adsorbing Cr in the environment [25]. It has been reported that Cr may be precipitated in the form of Cr_x_Fe_1-x_(OH)_3_ in soil [26]. Thus, the reductive dissolution of Fe(III) oxides promotes the release of structural binding Cr [27].

Microorganisms play an important role in the immobilization of heavy metals because they can promote the redox process of iron and affect the migration of metals [28]. For example, iron-reducing bacteria (FeRB) promote iron reduction to release related metals [29], so FeRB activity may affect the mobility and absorption of Cr [30]. Microorganisms also play a crucial role in the transformation and migration of Cr. Iron and sulfur-reducing bacteria are dominant in paddy soil [31]. *Bacillus* and *Anaeromyxobacter* have shown to be associated with Fe-Cr reaction [32,33]. Some scholars have pointed out that the reduction of Cr was essentially related to the microbial-mediated Fe(II)/Fe(II) cycle [34]. Understanding the role of rhizosphere microorganisms in Cr transformation and bioavailability is necessary to alleviate Cr pollution in rice. Therefore, it is necessary to determine the effect of water management on the composition and activity of rhizosphere soil microbial structure community.

At present, most of the research was mainly focused on the artificially added soil with high mobility and high toxicity of Cr(VI), but there are few studies on the effect of Cr availability on natural soil with high Cr geological background. In this study, we investigated the effects of different water management conditions on (1) the effect of Cr uptake by rice; (2) the distribution of Cr and Fe fractions in rhizosphere soil at different rice growth stages; and (3) the diversity and activity of microbial structure community in the rhizosphere soil. The results provide a scientific basis for the agronomic management practices of Cr-contaminated paddy fields in high geological background areas.

## 2. Materials and Methods

### 2.1. Pot Experiment

The experimental soil was collected from a paddy field in Maichen Town, Xuwen County, Zhanjiang City, Guangdong Province. The soil texture was loamy clay (40% sand, 28% silt, 32% clay), and the total Cr content was 228 mg kg^−1^. Other soil physical and chemical properties are shown in Table 1.

Pot experiments were carried out in the net room of the South China Agricultural University in Guangdong Province. We had chosen the local variety seeds of hybrid rice named Guang 8 You 305 for cultivation. Rice seeds were sterilized with 10% H_2_O_2_ for 15 min and then soaked in warm water at 30 °C for 24 h. Then, the soaked seeds were evenly spread on a large petri dish with wet filter paper and germinated in the dark. About 2 days later, the germinated seeds were transplanted into quartz sand matrix for culture. The Hoagland nutrient solution was used for culture, and the nutrient solution was replaced every 3 days. When the seedlings grew to 3 leaves, the rice was transplanted into the pure nutrient solution system for a week and finally transplanted into the soil pot.

The pot experiment included four growth stages (tillering stage, jointing stage, booting stage and filling stage) and two water management systems (continuous flooding (CF)) and alternating wet and dry (AWD)). Each treatment set up three repeats, a total of 24 pots. The water management methods are as follows: CF is to keep flooding to the liquid level of 5 cm during the whole growth period of rice; AWD means that the soil surface is flooded to 3 cm, and then the water is naturally dried, and the soil surface is dried for one day before flooding, so repeatedly. Each pot (diameter 25 cm, height 30 cm, bottom diameter 16.5 cm) had 5 kg soil added to it; 5 g compound fertilizer (C/N/P = 15/15/15) was added as base fertilizer; and 2 L water was added to submerge. Then, rice was transplanted on the 35th day of germination; 3 rice seedlings were transplanted in each pot.

### 2.2. Plants Analyses

Rice was collected at the tillering stage, jointing stage, booting stage and filling stage on the 27th, 63rd, 80th and 90th day after transplanting, respectively. The roots, stems and leaves of rice were separated, rinsed with water and then frozen in a refrigerator, followed by freeze-drying, crushing and digestion (HNO_3_:HClO_4_ = 4:1, *v*/*v*). The concentrations of total Cr and total Fe in plant samples were determined by flame atomic absorption spectrophotometer (FAAS, Z-2300, HITACHI, Tokyo, Japan).

### 2.3. Soil Analyses

Rhizosphere soil samples were collected with plants at different periods. One part of the collected soil was used for the determination of physical and chemical properties such as Cr content and morphological characteristics, and the other part was stored at −80°C in a refrigerator waiting for the determination of microbial community structure. The first part of the soil samples was dried, crushed and screened with 10 mesh and 200 mesh sieves. Cr fractions were extracted according to the sequentially extraction method proposed by Rinklebe [21]. In brief, weigh accurately 2 g soil and add the corresponding reagent in turn according to the order of extraction, then shake at room temperature (120rpm) and centrifuge (3000rpm, centrifuge for 10 min). Finally, the filtrate was filtered with 0.45 µm membrane and stored in a 4 °C refrigerator for test. After that, the extracted soil was washed with 10 mL ultrapure water and then shaken and centrifuged at room temperature for 15 min. After the washing solution was poured out, the next extraction was performed. The fractions and extraction of Cr are as follows: exchangeable fraction (F1) was extracted with 0.2 mol L^−1^ CaCl_2_ for 24 h; carbonate-bound fraction (F2) was extracted with 1.0 mol L^−1^ NH_4_OAc (pH 6.0) for 24 h; Fe/Mn oxide-bound fraction (F3) was extracted with 0.1 mol L^−1^ NH_2_OH-HCl + 1.0 mol L^−1^ NH_4_OAc (pH 6.0) for 0.5 h; organic matter-bound fraction (F4) was extracted with 0.025 mol L^−1^ NH_4_EDTA (pH 4.6) for 1.5 h; amorphous iron oxide-bound fraction (F5) was extracted with 0.2 mol L^−1^ ammonium oxalate (pH 3.25) for 4 h; crystalline iron oxide-bound fraction (F6) was extracted with 0.1 mol L^−1^ ascorbic acid +0.2 mol L^−1^ NH_4_-oxalate (pH 3.25) and shaken in a 95 °C water bath for 0.5 h; and the residual fraction (F7) was digested with HNO_3_-HClO_4_-HF [35,36]. The Cr concentration of the above digestion solution was determined by inductively coupled plasma optical emission spectrometer (ICP-OES, 710-ES, VARIAN, Palo Alto, CA, USA).

In addition, the extraction of different fractions of iron referred to the method proposed by Yu et al. [37]. The fraction includes dissolved-Fe, hydrochloric acid extractable iron (HCl-Fe), sulfite-citric acid-sodium bicarbonate extractable iron (DCB-Fe) and amorphous iron (Oxalate-Fe). The extraction method of each component iron is as follows: add 20 mL ultrapure water (pH = 7.0) to 1 g soil and shake for 16 h, then centrifuge at 4500rpm for 30 min, filter, and collect filtrate to determine the concentration of dissolved-Fe; add 25 mL 0.5 M HCl to 0.5 g soil, shake for 4 h, filter and collect the filtrate to determine the concentration of HCl-Fe. The extraction method of disulfite-citric acid-sodium bicarbonate (DCB) extractable Fe is by adding 1.0 g soil to a 50 mL polyethylene centrifuge tube and adding 20 mL 0.3 M trisodium citrate and 0.1 M sodium bicarbonate. The mixed solution was shaken at 120 rpm for 10 min in a 75 °C water bath, and then 1.0 g of sodium disulfite was added. After shaking for 5 min, 1.0 g of sodium disulfite was added again and then shaken for 10 min. Finally, the mixture was centrifuged at 2500rpm for 5 min, and then the supernatant was collected for determination. Amorphous iron (oxalate-Fe) was added to 0.5 g soil in a 50 mL polyethylene centrifuge tube; 25 mL oxalic acid-ammonium oxalate buffer solution (0.2 M ammonium oxalate pH 3.0) was added then shaken at 25 °C for 4 h and then filtered to determine Fe concentration. Finally, about 0.1 g soil samples were weighed and digested with HNO_3_-HClO_4_-HF to determine the total iron concentration. The iron content in the above extract were analyzed by atomic absorption spectrometer (FAAS, Z-2300, HITACHI, Tokyo, Japan).

### 2.4. Microbial Diversity Analyses

The rhizosphere soil samples stored at −80 °C were used for microbial community diversity analysis. The formal PCR test used transgenic AP22-02: Trans Start Fastpfu DNA Polymerase, 20 μL reaction system.

A set of primers (forward primer: 338F (ACTCCTACGGGAGGCAGCAG) and reverse primer 806R (GGACTACHVGGGTWTCTAAT)) were used for amplification. According to the preliminary quantitative results of electrophoresis, the PCR products were detected and quantified by QuantiFluor TM-ST blue fluorescence quantitative system (Promege, Paris, France), and then the Illumina library was constructed and sequenced. The final data were analyzed on the online platform of Majorbio cloud platform (www.majorbio.com, accessed on 5 October 2022).

### 2.5. Quality Control and Statistical Analyses

Quality control and assurance measurements for all analytes were performed using method blanks, triplicates and certified reference materials. GBW07405(GSS-5) (Beijing, China) and GBW07443(GSF-3) (Beijing, China), obtained from the National Standard Material Centre of China, were used as the standard materials for metals and fraction of Cr analyses. Recovery rates of reference materials ranged from 91% to 106% and from 95 to 109% for total Cr and fraction of Cr in soil samples, respectively. The sums of relative proportions of the 7 fractions were in the range of 89–105%, indicating that the Cr contents of sequential extraction were in an acceptable range. GBW10020(GSB-11) served as standard sample for plant samples. Recovery rates were ranged from 95% to 101%.

The data were analyzed with IBM SPSS Statistics 25.0 (IBM Corp., Armonk, NY, USA) software. The significant differences among the different treatment means were determined by one-way ANOVA analysis at *p* < 0.05. All data are presented as means ± standard error (SE) (*n* = 3). Redundancy analysis (RDA) between microbial community samples and soil environmental factors was performed on the microbial Majorbio cloud platform (software: R language vegan package), and spearman correlation coefficient was also used for correlation Heatmap analysis (software: R (version 3.3.1) pheatmap package).

## 3. Results and Discusses

### 3.1. Biomass of Rice Plant

Dry biomass reflects the resistance of plants to different soil conditions. The dry weight of each part of plant increased with the growth of rice (Table 2). At the same time, the dry weight of each part of the rice in CF was larger than AWD. The dry weight of rice roots, stems and leaves in CF treatment increased from 1.12 g, 1.72 g and 1.82 g to 4.6 g, 23.71 g and 13.2 g, respectively. The dry weight of roots, stems and leaves treated with AWD ranged from 1.08–2.27 g, 1.5–14.0 g and 1.49–9.38 g, respectively. This indicated that AWD treatment inhibited the growth of rice. This is consistent with the results of a previous study that the grain yield, plant height and root biomass of rice under AWD conditions are lower than CF conditions [38]. Studies have shown that AWD treatment at low Cr rates may promote rice growth by increasing the content of available nitrogen and organic matter in the soil during AWD, while inhibiting rice growth at high Cr rates [17]. Thus, the growth inhibition may be due to the increase in Cr toxicity [39]. In contrast, other literature reported that AWD irrigation increased the grain yield of rice. The differences between these studies may be due to differences in the timing of irrigation methods and basic physical and chemical properties of soils.

### 3.2. The Uptake and Translocation of Cr by Rice Plants

**Figure 1:**
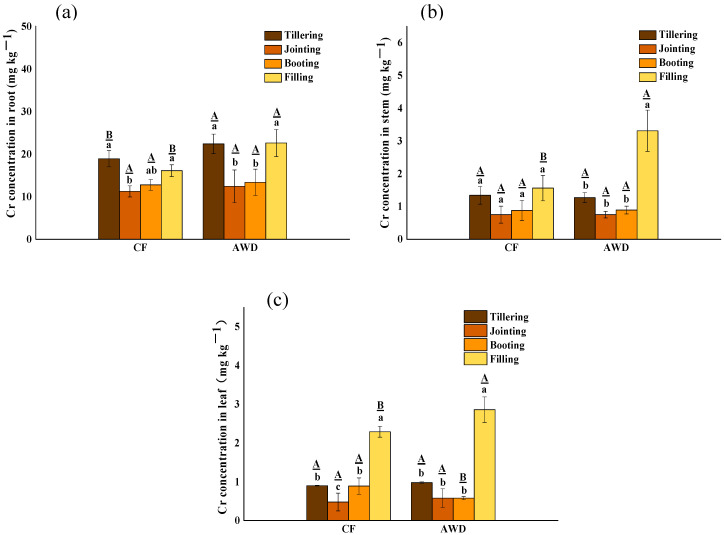
Effects of different water managements on Cr concentrations in roots (**a**), stems (**b**) and leaves (**c**) of rice at different growth stages. The lowercase letters indicate the differences among the same treatment in different periods; the uppercase letters indicate the differences between different treatments in the same period, *p* < 0.05.

As shown in Figure 1, the content of Cr in rice plants under CF treatment showed root (11.24–18.91 mg kg^−1^) > stem (0.75–1.56 mg kg^−1^) > leaf (0.48–2.29 mg kg^−1^), and AWD treatment also showed root (12.43–22.60 mg kg^−1^) > stem (0.81–3.31 mg kg^−1^) > leaf (0.58–2.86 mg kg^−1^), similarly. This is consistent with the distribution order of Cr in different parts of most plants: root > stem > leaf [40,41,42]. The Cr content of rice at the tillering stage and filling stage was higher than that at the jointing stage and booting stage, and there were significant differences among different growth stages. The high Cr content of rice at the tillering stage may be due to the rapid absorption of nutrients and relatively small biomass. With the growth of the plant, the biomass of rice is no longer growing rapidly, but the accumulation of Cr in rice continues. During the filling stage, there is a drainage period and an obvious oxidation process occurs. When the environmental conditions change from anaerobic to aerobic, Fe(II) oxidation can form to Fe(III)(hydride) oxides, which are the ubiquitous adsorbents of heavy metals (such as Cr). During oxidation, a large number of protons are released and the pH is significantly reduced. The complexation of Cr(III) and Fe(III/II) with NOM may result in the formation of Cr(III)-NOM-Fe colloids that enhance the fluidity of Cr(III). In addition, oxidation of S^2−^ to SO_4_^2−^ may contribute to Cr release [21].

There was a significant difference in rice tissues between different water managements. The Cr content in AWD treatment was higher than CF; the roots, stems and leaves were exceeded by 40%, 89% and 25% in the filling stage, respectively. This indicated that AWD treatment promoted the absorption and translocation of Cr by rice plants, which was consistent with the previous conclusion that AWD inhibited the biomass of rice plants. Studies had shown that irrigation has a significant effect on Cr content in rice tissues under AWD treatment with high soil Cr concentration [17]. Compared with CF treatment, soil conditions changed alternately from reduction to oxidation during rice growth. The AWD treatment reduced the reduction rate of Cr (VI) and increased the content [43]. Therefore, despite the high Cr geological background area with low Cr(VI) content, AWD could also increase the absorption of Cr by rice. However, the situation is different between different elements. In contrast, arsenic (As) and Cr have the opposite results. For As, maintaining aerobic conditions for rice growth can significantly reduce As uptake by rice [44]. The water in the soil was usually dominated by As(V) rather than the highly soluble and toxic As(III) under oxidizing conditions [45]. The phenomenon may be affected by the transformation of heavy metal bioavailable fraction in the soil.

### 3.3. Effects of Water Managements on Concentration and Fractions of Cr and Fe in Soil

**Figure 2:**
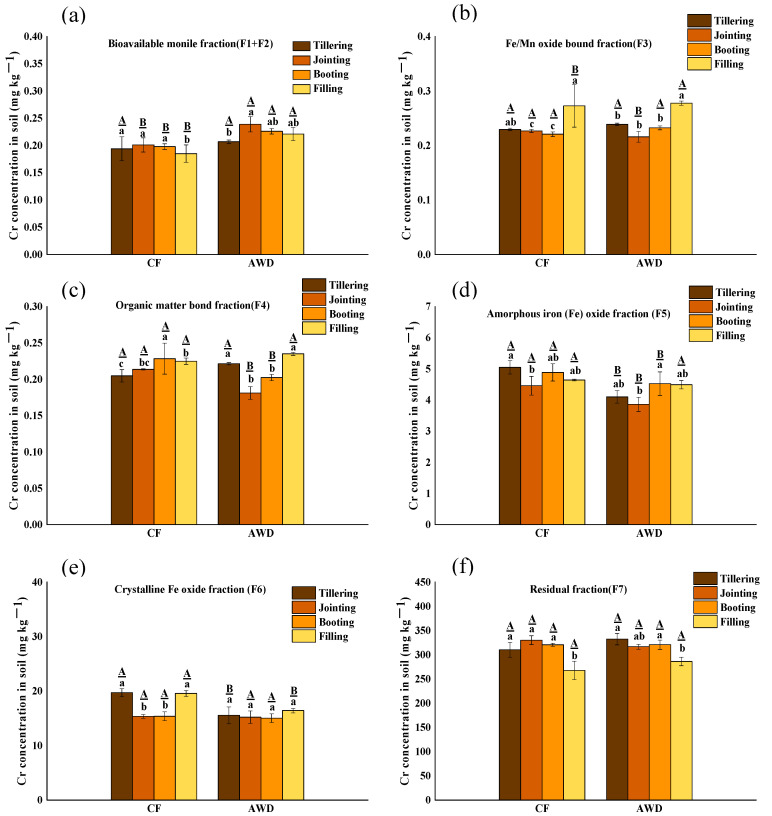
The concentration distribution of bioavailable fraction (F1+F2) (**a**), Fe/Mn oxide-bound fraction F3 (**b**), organic matter-bond fraction F4 (**c**), amorphous iron oxide fraction F5 (**d**), crystalline Fe oxide fraction F6 (**e**) and residual fraction F7 (**f**) of Cr in soil under different water management treatments at different growth stages. The lowercase letters indicate the differences among the same treatment in different periods; the uppercase letters indicate the differences between different treatments in the same period, *p* < 0.05.

In order to better understand the mobility of Cr in paddy fields, the changes of Cr fractions in different periods and different water management were sequentially extracted (Figure 2). In general, the Cr fraction distribution is F7 (residual fraction) > F6 (crystalline iron oxidation fraction) > F5 (amorphous iron oxide fraction) > F4 (organic matter-bound fraction) > F3 (Fe/Mn oxide-bound fraction) > F2 (carbonate-bound fraction) > F1 (exchangeable fraction). Residual fraction Cr (F7) is the main component of Cr in soil, accounting for more than 90%, indicating that the mobility of Cr is low [46]. In the four growth stages, the concentrations of bioavailable fractions ∑(F1–F2) in CF and AWD were 0.19–0.20 mg kg^−1^ and 0.20–0.20 mg kg^−1^, respectively, accounting for only 0.06–0.07% of total Cr. Interestingly, the CF and AWD treatments had significant differences between the bioavailable fractions ∑(F1-F2) and potential bioavailable fractions ∑(F3–F6), respectively. For CF treatment, the ∑(F1-F2) fraction and Fe/Mn oxide-bound fraction concentrations were decreased significantly with rice growth, and organic-bound Cr and residual Cr were increased significantly. This result indicated that the Cr fraction in soil was affected by Fe-Mn oxides in a strong redox state under flooding conditions. This result was in accordance with the latest research [47] that newly released Cr may be re-adsorbed on the surface of minerals and organic matter through electrostatic or physical complexes, resulting in a decrease in bioavailable Cr. However, it is different in AWD treatment; the ∑(F1–F2) fraction and Fe/Mn oxide-bound fraction showed a significant increase with rice growth. In contrast, the organic-bound and residual Cr fraction concentration were significantly reduced with rice growth. It is indicated that organic-bound Cr and residual Cr are significantly reduced and converted into Fe/Mn oxide-bound Cr and bioavailable Cr. The reason could be due to the oxidation of iron, because the oxidation of Fe(II) produces amorphous iron hydrates with poor crystallinity [48]. In summary, it can be seen that compared with CF treatment, AWD treatment increased the content of bioavailable and potential bioavailable component Cr in the soil and increased the absorption of Cr by rice.

CF treatment usually results in oxygen depletion, leading to anoxic fermentation of organic matter using various alternative electron acceptors (e.g., iron oxide, NO_3_^−^ and SO_4_^2−^) [49]. At the same time, a large number of protons are consumed, and pH is significantly increased, which may promote the adsorption of Cr(III) on the soil surface. In addition, organic matter (OM) may significantly affect the morphologic transformation of Cr. OM can be adsorbed on iron oxides to form organic-mineral iron oxides [50]. During flooding, reductive dissolution of Fe(III) (hydr) oxides results in the release of dissolved organic matter (DOM) from Fe(III) (hydr) oxides [6,51]. DOM contains rich active functional groups, which can form complexes with heavy metals [52,53]. Thus, DOM, such as low molecular weight DOM, may increase the mobility of Cr in paddy soils, resulting in significant dissolution of Cr(III) from Cr(III)-loaded goethite [54]. In addition, under flooding conditions, SO_4_^2−^ reduces to S^2−^, which may contribute to the fixation of Cr, as metals combine with S^2−^ to form metal sulfide precipitates, resulting in reduced solubility [55,56,57].

**Figure 3:**
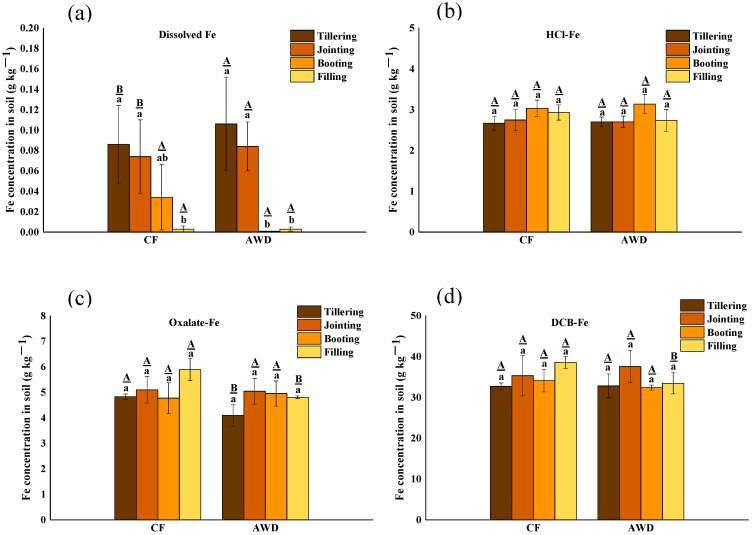
The concentration distribution of different fractions of Fe in soil under different water management treatments at different growth stages of rice. The lowercase letters indicate the differences among the same treatment in different periods; the uppercase letters indicate the differences between different treatments in the same period, *p* < 0.05.

The distribution of Fe fraction concentration in soil is shown in Figure 3. There was a significant difference in soil-dissolved Fe among different periods, and it decreased significantly with the growth period. The concentration of dissolved-Fe in AWD treatment was higher than that in CF treatment at the tillering stage and booting stage but lower in the other two stages. It may be the reason that dissolved-Fe was actively utilized by microorganisms under AWD treatment. There was no significant effect on the change of other Fe fraction concentration with CF treatment and AWD treatment. HCl-Fe is thought to be mainly derived from microbial reduction of poorly crystalline ferric hydroxide(III) or poorly ordered ferric hydroxide(III). Thus, the amount of extractable Fe(II) with hydrochloric acid can be used to assess the ability of microorganisms to reduce low crystalline Fe(III) hydroxide. The concentration of extractable Fe(II) in hydrochloric acid increased significantly in both treatments. For DCB-Fe, it is considered to be free iron oxides, both crystalline and low-crystalline. Similar to HCl-Fe, the concentration of DCB-Fe increased significantly after tillering. The reason may be that less O_2_ was released from the roots after the tillering stage of rice compared to the previous stage, resulting in reduced oxidation of Fe(II), and strong reducing conditions led to reductive dissolution of Fe(III) (hydrodr) oxides [58].

During different redox conditions, iron biogeochemical cycles in paddy soils, including Fe(III) reduction and Fe(II) catalyzing recrystallization of Fe(III)(hydr)oxides, have a significant influence on the immobilization of heavy metals [58]. Strong reducing conditions lead to reductive dissolution of Fe(III) (hydr)oxides, which play a crucial role in controlling the mobility of metals and may have a large effect on the release of Cr. The presence of Fe(II) can promote the recrystallization of metastable Fe(III)(hydr) oxides, resulting in the incorporation of Cr. For example, ferrites can be converted into more stable goethite [59]. These transformations of Fe(III)(hydr) oxides may significantly affect the mobility and availability of Cr in paddy soils.

### 3.4. Microbial Community Analyses

**Figure 4:**
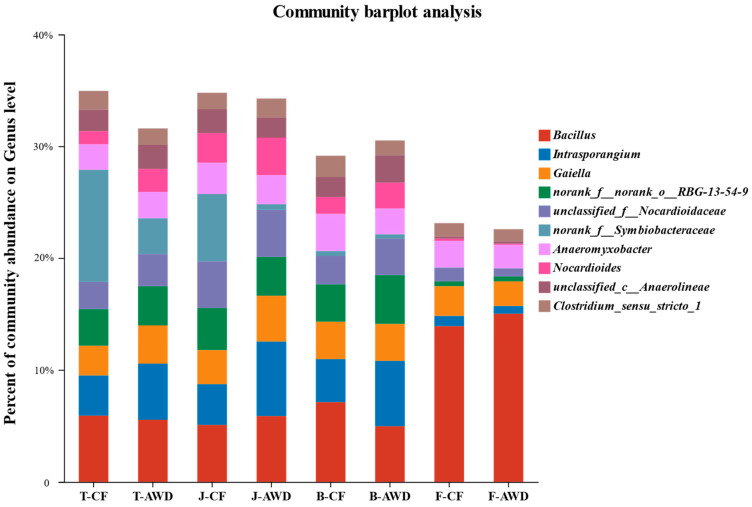
The relative community abundance of the top 10 soil bacteria on genus level in different treatments. T represents the Tillering stage; J represents the Jointing stage; B represents the Booting stage; F represents the Filling stage. CF refers to continuous flooding. AWD refers to alternate wet and dry irrigation.

The composition and abundance of microbial community are important factors affecting the migration and bioavailability of heavy metals in paddy soils. Rhizosphere soil microorganisms can maintain soil fertility and plant yield by assisting nutrient fixation, mineralization, decomposition and flow [60,61]. In this study, a total of 50 phyla, 163 classes, 373 orders, 566 families, 1052 genera and 2084 species were identified. Figure 4 describes the relative community abundance of microbial groups at the genus level in different water managements at different growth stages of rice. At genus level, the microbial community composition was similar, but the relative abundance was different. Among them, Bacillus (3.7–11.6%) accounted for the largest proportion, followed by Intrasporangium and Gaiella. Compared with other growth stages, the soil at the filling stage significantly increased the abundance of Bacillus and decreased the abundance of Intrasporangium. Bacillus is known as Fe (III)-reducing genus, which is speculated to be related to Fe-Cr reaction in previous studies [62]. It indicates that the transformation of Cr in soil is closely related to microbial-mediated iron cycle [34]. Clostridium was found to exist in CF treatment (1.5–1.8%) and AWD treatment (1.2–1.6%) at the genus level, which is a typical sulfate-reducing bacteria related to sulfur metabolism [63,64]. It can reduce sulfate to sulfide and provide an electron donor for the conversion of Cr.

**Figure 5:**
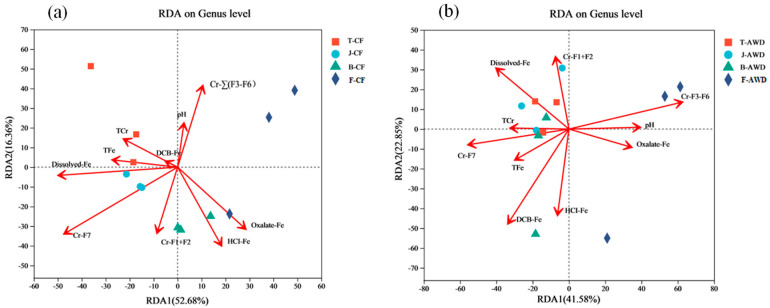
The redundancy analysis (RDA) between bacterial community and related environmental variables of continuous flooding treatment (CF) (**a**) and alternative wet and dry treatment (AWD) (**b**) at genus level. TCr and TFe represent the Total Cr and the Total Fe, respectively; Cr-F1+F2 represent the bioavailable fractions of Cr; Cr-∑(F3–F6) represent potentially bioavailable fractions of Cr; Cr-F7 represents the residual fractions of Cr. (T represents the Tillering stage; J represents the Jointing stage; B represents the Booting stage; F represents the Filling stage.).

In this paper, redundancy analysis (RDA) was used to determine the correlation between soil environmental variables and soil microbial community composition (Figure 5). RDA analysis showed that at the genus level, the relevant environmental variables explained 69.04% and 64.43% of the community composition of CF treatment (Figure 5a) and AWD treatment (Figure 5b), respectively. Compared with other environmental variables, dissolved-Fe (*p* = 0.048), Cr-F7 (*p* = 0.015) and Cr-∑(F3–F6) (*p* = 0.096) had significant effects on microbial community composition. Dissolved-Fe (RDA1 = −99.85%, r^2^ = 0.50, *p* = 0.048) and Cr-F7 (RDA1 = −85.21%, r^2^ = 0.64, *p* = 0.015) were significantly negative related with RDA1, while TFe (RDA1 = −99.07%, r^2^ = 0.15, *p* = 0.484), TCr (RDA1 = −87.01%, r^2^ = 0.14, *p* = 0.533) and DCB-Fe (RDA1 = −88.22%, r^2^ = 0.01, *p* = 0.989) were critical for explaining the variations in community structure in CF treatment. The soil pH (RDA1 = 99.18%, r^2^ = 0.17, *p* = 0.455), Cr-∑(F3–F6) (RDA1 = 99.48%, r^2^ = 0.45, *p* = 0.096), TCr (RDA1 = −98.53%, r^2^ = 0.12, *p* = 0.571) and Cr-F7 (RDA1 = −99.99%, r^2^ = 0.35, *p* = 0.140) were critical factors explaining the variations communities in AWD treatment. Some scholars have shown that soil moisture has the greatest impact on the composition of microbial communities in the 0 cm surface soil, and pH may be the main reason for the stratification of the 20 cm surface soil. In addition, the composition of microbial communities below 40 cm is mainly significantly affected by total Cr [53]. In the RDA plot, the angle between the 2 factor vectors is less than 90°, indicating a positive correlation between them. It can be seen from Figure 5a that TCr is positively correlated with TFe and dissolved-Fe under CF condition, while TCr is positively correlated with TFe, Cr-F7 and dissolved-Fe under AWD condition. It shows that the Cr content in the soil is greatly affected by the effective state of iron. Similarly, the RDA results of Xiao et al. [65] also showed a close correlation between microbial community composition and soil pH and Fe(II) concentration.

**Figure 6:**
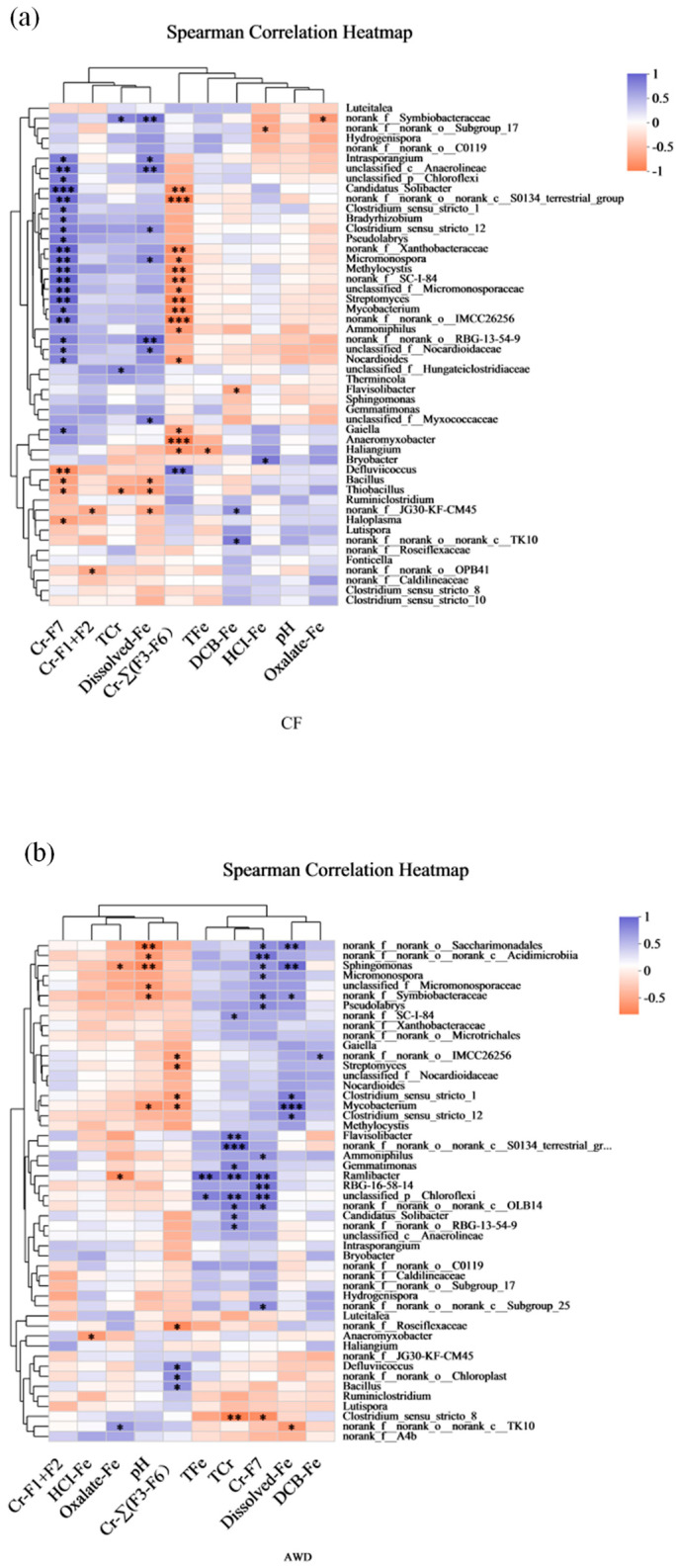
The spearman correlation on genus level between bacterial community and related environmental variables of continuous flooding treatment (CF) (**a**) and alternative wet and dry treatment (AWD) (**b**). TCr and TFe represent the Total Cr and the Total Fe, respectively; Cr-F1+F2 represent bioavailable fractions of Cr; Cr-∑(F3–F6) represent the potentially bioavailable fractions of Cr; Cr-F7 represents the residual fractions of Cr. (* means 0.01 ≤ *p* ≤ 0.05, ** means 0.001 ≤ *p* ≤ 0.01, and *** means *p* ≤ 0.001).

Spearman correlation analysis was further used to study the relationship between dominant species and soil environmental factors (Figure 6). The results showed that Cr-F7 (*p* < 0.001), dissolved-Fe (*p* < 0.01) and Cr-∑(F3-F6) (*p* < 0.001) were significantly and specifically correlated with multiple soil microbial communities in CF treatment, followed by Cr-F1+F2, TCr, TFe, DCB-Fe and Oxalate-Fe (*p* < 0.05), and the dominant species had little correlation with pH. It shows that different forms of Cr and different forms of iron have obvious ecological effects on soil microbial communities. In AWD treatment, pH (*p* < 0.01), TCr (*p* < 0.001), Cr-F7 (*p* < 0.01) and dissolved-Fe (*p* < 0.001) were significantly correlated with multiple microbial communities, respectively. Except for Cr-F1+F2, there was significant correlation between microbial communities and other environmental factors (*p* < 0.05). Among these bacteria, *Anaeromyxobacter*, another functional Fe(III)-reducing bacterium, has also been speculated to be related to Fe-Cr reactions in previous studies. *Anaeromyxobacter* was positively correlated with Cr-∑(F3–F6) in CF treatment. In contrast, *Anaeromyxobacter* was significantly negatively correlated with HCl-Fe in AWD treatment. In addition to Fe-reducing bacteria, the relative abundance of sulfate-reducing bacteria was considerably higher in the AWD-treated soils than CF, with *Clostridium* being the most dominant. In CF treatment, *Clostridium* was significantly positively correlated with dissolved-Fe and Cr-F7. In contrast, *Clostridium* was significantly positively correlated with Cr-∑(F3–F6) and dissolved-Fe in AWD treatment. It is particularly noteworthy that there is a significant difference in the correlation between pH value and microbial community between AWD treatment and CF treatment, indicating that AWD treatment may affect the difference of microbial community by affecting soil pH, thus affecting the migration and transformation of Cr. Previous studies have also shown that soil pH can affect the composition and activity of soil microbial communities [66]. Changes in pH affect the physiological and biochemical processes of heavy metals and cell bodies and ultimately affect the survival and growth of specific bacteria, resulting in changes in microbial community structure [67]. In conclusion, it can be clear that the effect of AWD treatment on Cr in a rice–paddy soil system has microbial factors.

## 4. Conclusions and Environmental Implications

Different water managements had significantly different effects on the migration and transformation of Cr in a rice–paddy soil system. Alternative wet and dry (AWD) treatment made Cr bioavailable in the soil and also affected the soil microbial community of iron-reducing microorganisms and sulfate-reducing bacteria, which provided electron donors for Cr transformation, thus affecting the migration and transformation of Cr in soil. AWD treatment promoted the absorption of Cr by rice and affected its biomass. The reason for this result may be that Cr is released through reduction of Fe(III) (hydr) oxides, and Cr is likely to enter the structure of Fe(III) (hydr) oxides through Fe(II)-catalyzed recrystallization. Basalt is an important Cr reservoir in the terrestrial environment. Cr in paddy soil can be enriched by chemical weathering. Basalt-derived paddy soils contain very high concentrations of Cr and can enter the human body through the food chain. Therefore, the fluidity and availability of Cr are critical to the risk assessment in basaltic paddy soils. This study revealed the variation of Cr availability and mobility in different water management processes of basalt-type paddy soils, which provided a basis for formulating effective water management strategies to control Cr conversion in basalt-type paddy soils.

## Figures and Tables

**Table 1:** The basic physical and chemical properties of soil.

Properties	Soil
pH	5.74
Total Cr (mg kg^−1^)	228
Total Fe (g kg^−1^)	76.84
Organic matter (g kg^−1^)	30.21
Cation exchange capacity (cmol kg^−1^)	25.16
Total N (g kg^−1^)	2.121
Soil texture	40% sand, 28% silt, and 32% clay

**Table 2:** Biomass of rice plants in different water management at four growth stages.

Dry Weight (g)		CF	AWD
root	Tillering	1.12 ± 0.05 a	1.08 ± 0.01 a
	Jointing	2.06 ± 0.35 a	1.78 ± 0.26 b
	Booting	3.07 ± 0.42 a	1.89 ± 0.18 b
	Filling	4.60 ± 0.57 a	2.27 ± 0.38 b
stem	Tillering	1.72 ± 0.43 a	1.500 ± 0.02 a
	Jointing	6.42 ± 0.45 a	5.66 ± 0.13 b
	Booting	3.07 ± 0.42 a	10.74 ± 0.77 b
	Filling	23.71 ± 1.44 a	14.00 ± 0.85 b
leaf	Tillering	1.82 ± 0.56 a	1.49 ± 0.05 a
	Jointing	5.04 ± 0.47 a	4.67 ± 0.43 b
	Booting	5.67 ± 1.34 a	4.68 ± 0.56 b
	Filling	13.20 ± 1.05 a	9.38 ± 0.46 b

The lowercase letters indicate the difference between different treatments in the same period, *p* < 0.05. CF refers to continuous flooding. AWD refers to alternate wet and dry irrigation.

## Data Availability

Not applicable.
